# How VEGF-A and its splice variants affect breast cancer development – clinical implications

**DOI:** 10.1007/s13402-022-00665-w

**Published:** 2022-03-18

**Authors:** Hivin Al Kawas, Inas Saaid, Paul Jank, Christina C. Westhoff, Carsten Denkert, Therese Pross, Karoline Barbara Stephanie Weiler, Maria Margarete Karsten

**Affiliations:** 1 Department of Gynecology with Breast Center, Charité – Universitätsmedizin Berlin, Corporate Member of Freie Universität Berlin, Humboldt-Universität zu Berlin, Charitéplatz 1, 10117 Berlin, Germany; 2 Institute of Pathology, Philipps-Universität Marburg, 35043 Marburg, Germany; 3 Clinic for Obstetrics and Gynaecology Dritter Orden, Menzinger Str. 44, 80638 Munich, Germany

**Keywords:** Breast cancer, VEGF, Angiogenesis, Vascular endothelial growth factor, Splice variants, VEGF_165_b

## Abstract

**Background:**

Altered expression levels and structural variations in the vascular endothelial growth factor (VEGF) have been found to play important roles in cancer development and to be associated with the overall survival and therapy response of cancer patients. Particularly VEGF-A and its splice variants have been found to affect physiological and pathological angiogenic processes, including tumor angiogenesis, correlating with tumor progression, mostly caused by overexpression. This review focuses on the expression and impact of VEGF-A splice variants under physiologic conditions and in tumors and, in particular, the distribution and role of isoform VEGF_165_b in breast cancer.

**Conclusions and perspectives:**

Many publications already highlighted the importance of VEGF-A and its splice variants in tumor therapy, especially in breast cancer, which are summarized in this review. Furthermore, we were able to demonstrate that cytoplasmatic VEGFA/_165_b expression is higher in invasive breast cancer tumor cells than in normal tissues or stroma. These examples show that the detection of VEGF splice variants can be performed also on the protein level in formalin fixed tissues. Although no quantitative conclusions can be drawn, these results may be the starting point for further studies at a quantitative level, which can be a major step towards the design of targeted antibody-based (breast) cancer therapies.

## Introduction

In a variety of physiological and pathological processes, angiogenesis plays an important role in the formation of new capillary blood vessels, thereby enabling tissue growth and repair. In normal tissues these processes are kept in balance (homeostasis) by pro- and anti-angiogenic factors. In diseased tissue such as cancer tissue, however, dysregulation leads to imbalance. This imbalance occurs as a result of an increased metabolic demand of the tumor and a higher vascularization required. Vascular endothelial growth factors (VEGFs), in particular VEGF-A, have been identified as key factors for inducing tumor angiogenesis. Here, we aim to provide an overview of the characteristics of VEGF, its regulation and overexpression, as well as the importance of its splice variants and their pro- and anti-angiogenic roles.

## VEGF and its major characteristics

Angiogenesis is regulated both spatially and temporally by coordinated interactions between activators and inhibitors. One potent pro-angiogenic factor is vascular endothelial growth factor (VEGF), which not only plays an important role during embryonic development, but also in adult organisms [1]. The most obvious effect of VEGF is the formation of new vessels stimulated by hypoxia [2], but also by upregulated factors like cytokines [3–5], hormones such as progesterone [6] and testosterone [7] and transcription factors such as c-Fos [8]. Also other processes may be induced by VEGF, such as proliferation and migration, which primarily affect endothelial cells due to their high expression of VEGF receptors (VEGFRs) [9, 10], or inducing a pronounced biphasic increase in permeability, which is selective for small and medium sized molecules [11], and distinct vasodilation [12], caused by endothelial production of nitric oxide (NO), which in turn is stimulated by VEGF [13]. These numerous functions of VEGF can be explained by different cellular localizations of various subtypes of the tyrosine kinase receptors VEGFR-1, -2 and -3. While VEGFR-3 is primarily expressed on lymphatic endothelial cells, VEGRF-1 and -2 are not only expressed on endothelial cells, but also on neurons [14], hepatocytes [15], mast cells [16], hematopoietic stem cells [17], osteoblasts [18], retinal pigment epithelium cells [19, 20] and more. Endothelial cells also express NRP-1 (neuropilin-1) and NRP-2 (neuropilin-2), and act as isoform-specific receptors for VEGF [21]. Neuropilin was originally identified on neuronal cells as a receptor for the class 3 semaphorin/collapsin family of neuronal guidance mediators [22]. The diversity of VEGF-induced effects is also caused by the occurrence of different subtypes, including VEGF-A, and its different isoforms, VEGF-B, VEGF-C, VEGF-D, VEGF-E (viral VEGF), VEGF-F (snake venom VEGF) and placental growth factor (PlGF). Recently, the endocrine gland-derived vascular endothelial growth factor (EG-VEGF) has been added to this group [1]. Particularly VEGF-A, which was discovered as the first subtype, has been found to play an important role in both physiological and pathological angiogenic processes, including tumor angiogenesis, in which it correlates with tumor progression, mostly caused by overexpression of the growth factor. As a result, VEGF-A and its receptor VEGFR-2 have been considered as targets for various therapeutic approaches, not only for cancer, but also for other diseases such as diabetic retinopathy, diabetic macular edema and peripheral artery disease (PAD) [23, 24].

In the following chapters, current knowledge on VEGF-A expression, genetic variation, activation and clinical relevance in tumors, as well as modulators of VEGF-A that may be used as therapeutics for the treatment of diverse cancers, especially breast cancer and its different entities, are summarized.

## Physiological and pathological expression of VEGF-A

VEGF-A is secreted not only by endothelial cells [25–28], but also by other cells, in response to hypoxia, i.e., in tumor cells [25, 29], macrophages [25, 27, 28], platelets [28], keratinocytes [25, 28], kidney mesangial cells [25, 28], activated T-cells [25, 27, 28], leukocytes [26], dendritic cells [30], retinal pigmentary epithelial cells [31], Müller cells in the retina [32], astrocytes [25], osteoblasts [25], bronchial and alveolar epithelial cells [33], pericytes [34] and vascular smooth muscle cells (VSMCs) [35]. More recently, it has been found that VEGF-A is also expressed in myofibroblasts located in the myocardium, suggesting its implication in post-infarction tissue repair and remodeling [28]. Human VEGF-A contains eight exons separated by seven introns [36] and, by alternative mRNA splicing, creates different isoforms. Each isoform plays a specific role in the development and differentiation of the vascular system [27].

**Fig. 1:**
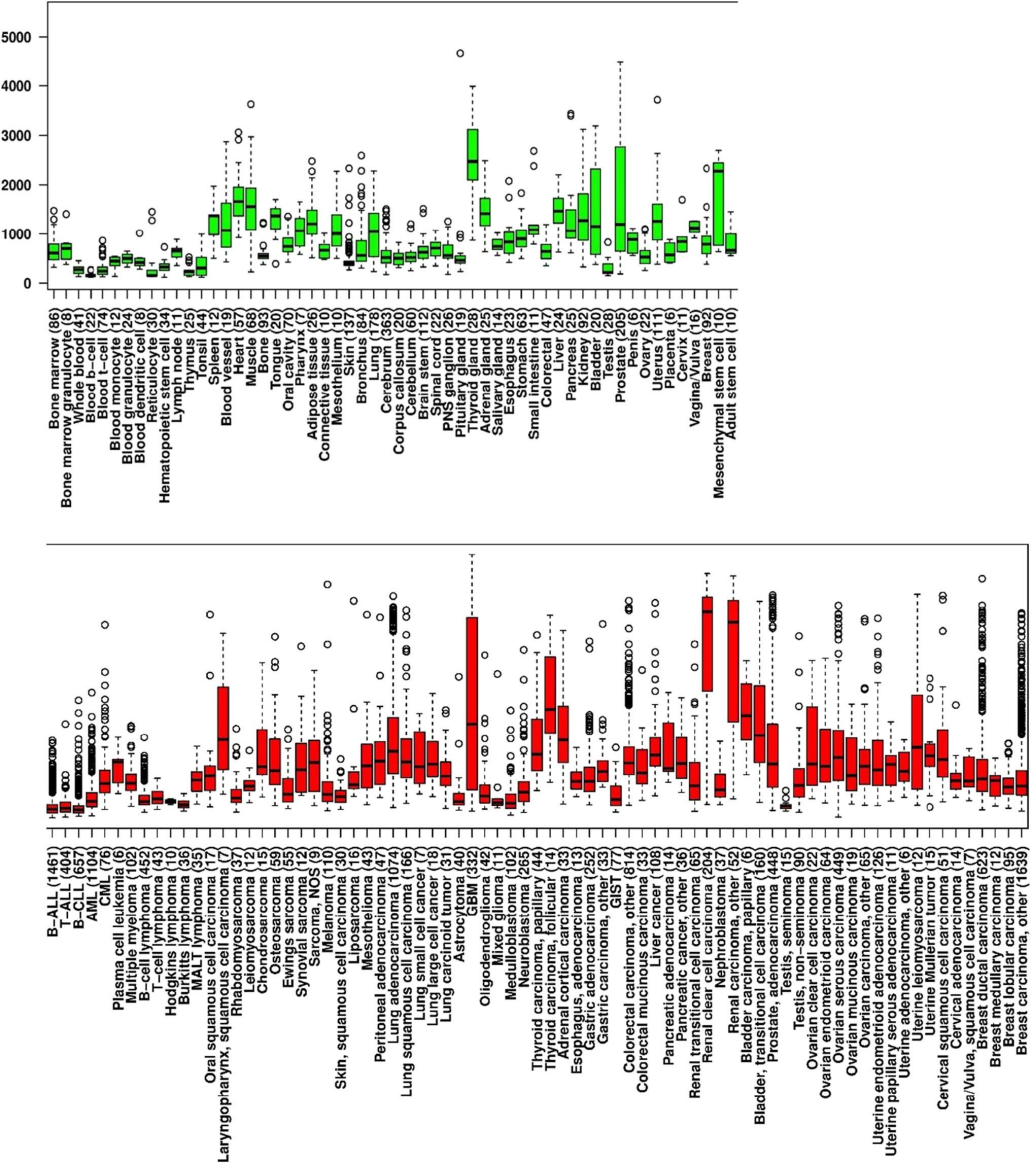
**VEGF-A expression in cancer patients.** The in silico transcriptomics database (http://ist.medis apiens.com/) was employed for VEGFA expression analysis in cancer and normal tissues (tissue boxplot). Green represents healthy tissues and red represents tumor tissues

Tumors require blood vessels to grow, which causes the production of pro-angiogenic factors by the tumor itself such as VEGF-A. This results in an “angiogenic switch”, whereby new vasculature is formed in and around the tumor, allowing it to grow exponentially. These blood vessels may be structurally abnormal, leaky and hemorrhagic, leading to a high interstitial pressure. As a result, the tumor blood flow is suboptimal, leading to hypoxia and further VEGF-A production. This is why it is overexpressed in many different types of cancer, including breast cancer (Fig. 1) [37–39].

## VEGF-A and its role in breast cancer

Several studies have shown that VEGF-A can induce tumor cell proliferation in mouse models of breast cancer [40]. Others have shown similar results in cats with breast cancer, where increased levels of VEGF-A, VEGFR-1 and VEGFR-2 were found especially in higher malignancy mammary carcinomas, such as HER2-positive and TN normal-like carcinomas [41]. Obermair et al. reported that intra-tumoral VEGF concentrations are significantly higher in breast cancer tissues than in fibromas or normal epithelial tissues of the breast [42]. Moreover, VEGF-A has been found to act as an autocrine survival factor for breast cancer cells [43–48]. VEGF-A blockade through VEGF neutralizing antibodies or siRNAs under normoxia and hypoxia resulted in direct tumor cell apoptosis [43–45]. In some studies, VEGF signaling has been shown to induce the survival of tumor cells through VEGFR-1 or VEGFR-2 [40, 47]. Targeted reduction of VEGFR-1 expression significantly decreased the survival of breast cancer cells through downregulation of protein kinase B (AKT) phosphorylation, while targeted reduction of VEGFR-2 or NRP1 expression had no effect on the survival of these cancer cells [47]. In addition to this, an autocrine loop has been found to exist for VEGF to induce breast cancer cell migration and/or invasion [49]. Thus, VEGF-A increases neovascularization and vasodilation or vessel maturation involving both blood vessels and tumor cells and acts by selective autocrine effects to stimulate tumor cell proliferation, survival, adhesion and chemotaxis. The existence of a distinct autocrine signaling loop is indicated by the production of VEGF-A by breast cancer cells and the activation of VEGF receptors at the surface of these cells, which enables them to promote their own growth, survival and migration by phosphorylation and activation of VEGFR-1/2 or VEGF-induced NRP signaling [50]. Other studies confirmed VEGF over-expression at both the protein and the mRNA levels [51], being markedly increased in human breast carcinomas but low in non-neoplastic tissues [52, 53]. VEGF mRNA has been found to be expressed more frequently in malignant breast cancer tissues than in non-tumorous breast tissues. As a result, a significant correlation of VEGF mRNA positivity with high vascular counts and positive axillary lymph nodes has been observed [53].

## Genetic regulation of VEGF

Post-transcriptional regulation of VEGF plays a significant role in its expression [54, 55]. The translation of most gene transcripts depends on interaction of ribosomes with a molecular "cap" at the 5' end of the UTR of mRNA [56]. This cap-dependent translation can be suppressed under cellular stress, such as hypoxia [54]. The guanine-cytosine-rich 5'-UTR of VEGF mRNA contains two internal ribosomal entry points (IRES) that initiate synthesis of the VEGF protein in a cap-independent manner [57, 58]. Small noncoding RNA sequences with an approximate size of 22 nucleotides, called microRNAs (miRNAs), have been found to play an important role in the post-transcriptional control of gene expression [59, 60]. They are also involved in the control of cell proliferation, apoptosis, cell cycle progression, migration and angiogenesis [61–63], by targeting the 3’UTRs of their target mRNAs. Binding to the mRNAs leads to translation inhibition or degradation [64]. It has been reported that several miRNAs can regulate vascular development, which is crucial for tumor development and progression [63, 65]. For example, miR-15b, miR-16, miR-20a and miR-20b have been found to act as potent anti-angiogenic miRNAs by targeting VEGF [66], while miR-379 and miR-874 exhibit different effects on tumor cell survival and growth [65, 67].

## VEGF-A overexpression in tumors

**Fig. 2:**
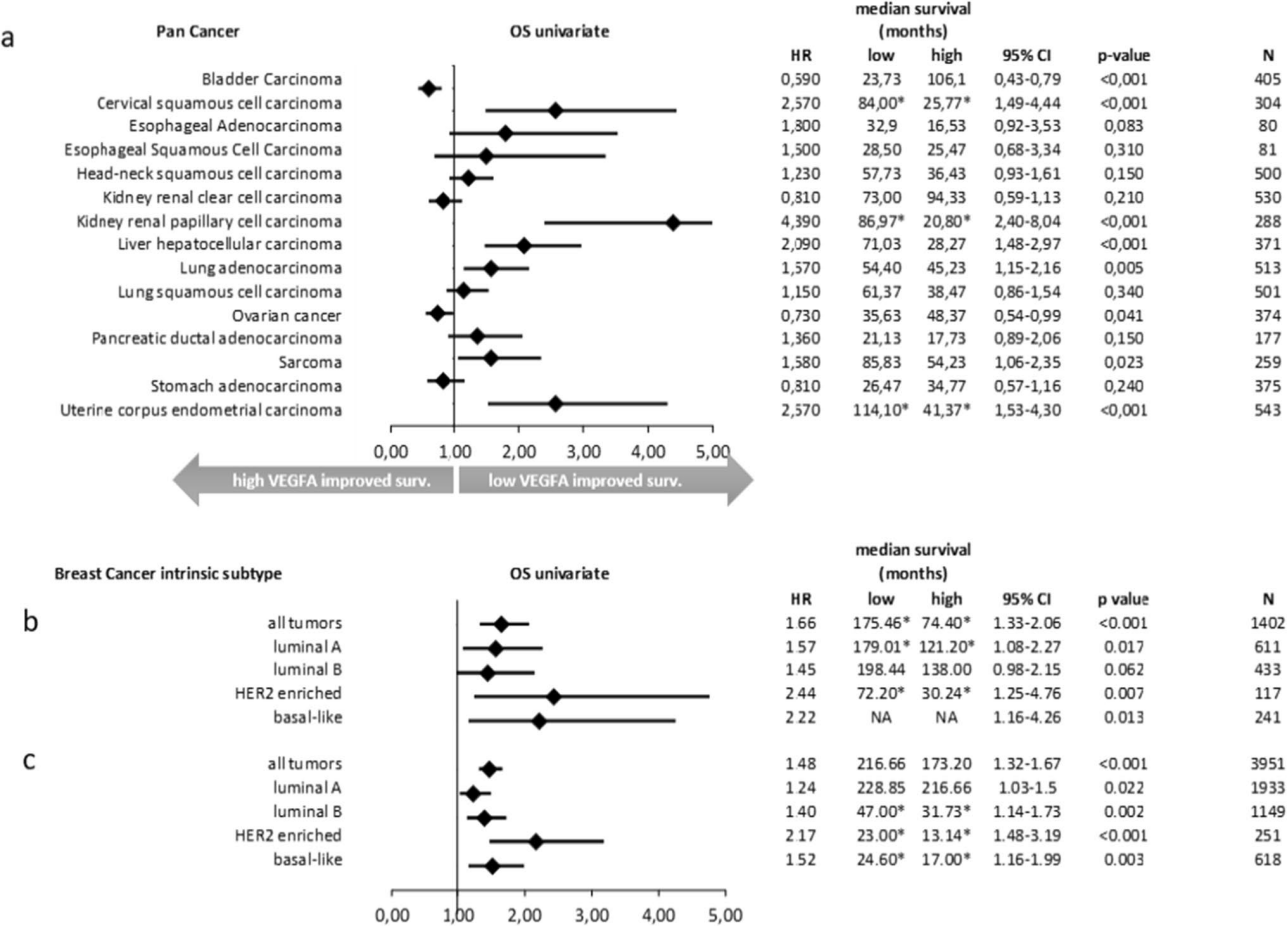
**Log-Rank test of continuous VEGF-A expression as a prognostic marker for recurrence-free survival (RFS) and overall survival (OS) and mean of OS & RFS**. (**a**) Hazard Ratio (HR) of OS from different cancer entities (pan cancer) (**b**) HR of OS from breast cancer and intrinsic subtypes (**c**) HR of RFS from breast cancer and intrinsic subtypes. Annotated numbers (*) refer to the upper quartile survival, since patients with these entities had an OS or RFS of over 50% within the given time period (120 months)

*VEGF* mRNA is overexpressed in the majority of human tumors and correlates with their invasiveness, vascular density, metastasis, recurrence and prognosis [29]. In numerous studies on the prognosis of breast cancer, micro-vessel density has been reported to affect the disease-free and overall survival of patients [68, 69]. By using the KMplot database (https://kmplot.com/analysis/), a link between VEGF-A mRNA expression and the overall survival (OS) of patients with different tumors and tumor subtypes was found (summarized in Fig. 2) [70]. Figure 2A shows OS based on VEGF-A mRNA expression in different tumor types as a forest plot. For example gynecological tumors, such as ovarian and endometrial carcinomas (uterine corpus), can thus be grouped with respect to OS, stratified by VEGF-A expression. Figure 2B and C are based on recurrence-free survival (RFS) and OS rates of breast cancer patients stratified by VEGF-A mRNA expression. As can be seen, all breast cancer subtypes benefit from low VEGF-A mRNA expression as a prognostic biomarker. Interestingly, HER2 enriched and triple-negative breast cancers (TNBC) benefit more from low VEGF-A expression with HR values of 2.44 and 2.22, respectively, with significant *p*-values (*p* = 0.007 and *p* = 0.0013). These results support a study of Howard et al., in which no significant correlation between HER2 overexpression and increased VEGF activity was found [71]. They hypothesized that the expression of VEGF is not regulated through HER2 in aggressive breast carcinomas, but through other mechanisms such as the expression of hypoxia-inducible factor 1 (HIF-1) in the absence of HER2 overexpression. This notion is based on a study from Zhong et al. in which it has been shown that HER2 stimulates VEGF via HIF-1 [72]. This study additionally showed that the α subunit of heterodimeric HIF-1 is activated during hypoxia or stimulation by growth factors and tumor-associated angiogenic factors such as VEGF [72, 73]. Laughner et al. also showed that HER2 signaling, induced by overexpression in mouse 3T3 cells or heregulin-β1 stimulation of human MCF-7 breast cancer cells, resulted in increased HIF-1α expression and, consequently, VEGF mRNA expression [74]. In comparison, luminal tumors were defined by HR = 1.57 (luminal A) and HR = 1.45 (luminal B) values. In almost all cancer studies to date, tumor suppressor genes are downregulated and tumor promoters (oncogenes) are overexpressed. Thus, in congruence with Figure 2, a low VEGF-A mRNA expression can have a beneficial effect on tumor development. It has already been shown by others that triple negative breast cancers show a higher EGFR expression [75], suggesting that in these cases tumor development is more dependent on growth factors when hormone receptors are not expressed. In comparison, the growth of luminal A and luminal B breast tumors is more controlled by hormone receptors, which also play important roles as treatment targets. This explains why HER2 enriched and triple negative breast tumors benefit more from lower VEGF mRNA expression levels, as shown below. In summary, blocking VEGF-A may be a therapeutic approach for HER2 enriched and triple-negative breast cancers.

## VEGF-A splice variants as key factors for physiological and pathological angiogenesis

**Fig. 3:**
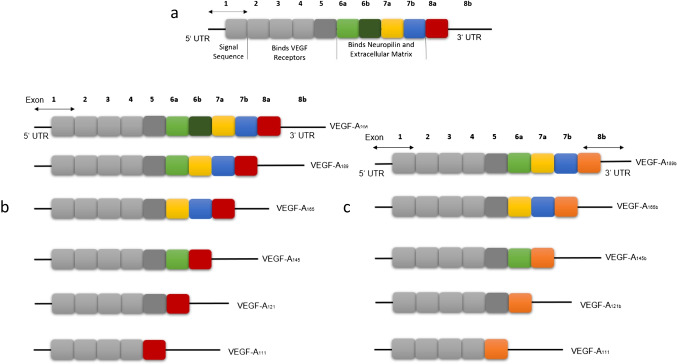
**Schematic illustration of the VEGF-A Gene, located on chromosome 6p21.1.** (**a**) It is structured in eight exons, separated by seven introns and generates alternative VEGF mRNAs by splicing. By selectively removing intron regions and joining specific combinations of exons, up to 16 different VEGF-Axxx isoforms are created. The xxx represent the number of amino acids present in the final protein sequence. The most common transcripts are VEGF_111_, VEGF_121_, VEGF_145_, VEGF_165_, VEGF_189_ and VEGF_206_. (**b**). Alternative splicing of the terminal exon, exon 8, gives rise to another isoform, anti-angiogenic VEGF-Axxxb, which has the same number of amino acids but different C terminal sequences (**c**)

The VEGF-A gene is located on chromosome 6p21.1 [76] and is composed of eight exons separated by seven introns [77]. It generates alternative VEGF mRNAs by splicing (Fig. 3). To this end, it selectively removes intron regions and joins specific combinations of exons, resulting in up to 16 different VEGF-Axxx isoforms. The xxx represent the number of amino acids present in the final protein sequence. The most common transcripts are VEGF_111_, VEGF_121_, VEGF_145_, VEGF_165_, VEGF_189_ and VEGF_206_ [78]. The domain encoded by exons 1-5 contains information required for recognition of the VEGF receptors KDR/flk-1 and flt-1 [79] and is present in all VEGF isoforms. The amino acids encoded by exon 8 are also present in all VEGF splice variants. Consequently, the peculiarity which distinguishes the VEGF isoforms is the presence or absence of peptide sequences encoded by exons 6 and 7 of the VEGF gene [80]. All of them, however, seem to induce endothelial cell proliferation and in vivo angiogenesis, in agreement with previous studies that have indicated that these functions are not dependent on the presence of either exon 6 or exon 7 [81] and seem to be associated with the ability to bind to the KDR/flk-1 receptor [82, 83], which all of them tested to date have been found to bind to [84]. VEGF_165_, the prevalent and most common isoform, plays a major role in stimulating endothelial cell proliferation and migration and binds to the co-receptors NRP-1 and NRP-2 [85]. It lacks the residues encoded by exon 6, thereby having moderate affinity for heparin and HSPG [77]. Thus, most of VEGF_165_ remains bound to the cell surface, whereas subtypes like VEGF_121_ lack the residues encoded by both exons 6 and 7 and thus have no affinity for heparin or HSPG, thereby existing in a free form [77, 86]. VEGF_165_ can promote the survival of motor neurons during hypoxia through binding to VEGFR-2 and NRP-1 [87], although elevated levels of VEGF_165_ have been linked to POEMS syndrome, also known as Crow-Fukase syndrome [88]. More importantly, together with VEGF_121_, it represents the most relevant inducer of tumor vascularization as it is overexpressed in various cancers, such as colon and lung cancers [89]. Furthermore, it exerts several effects in different pathways required in angiogenesis such as endothelial cell migration, proliferation, tube formation and survival [90] and is, therefore, subject to intense investigation. In addition, it has been found that VEGF_121_, although less abundant, is more mitogenic than VEGF_165_ or VEGF_189_ [91]. VEGF_111_ is encoded by exons 1–4 and is induced by DNA damage caused by ultraviolet B (UV-B) radiation and genotoxic drugs [92], as well as mild hypothermia [93]. It is not induced by hypoxia and hypoglycemia, unlike other VEGF isoforms [92], whereas under natural conditions it has been found to be expressed only in the uterine wall, testes and kidneys of *Saiphos equalis*, a viviparous lizard from eastern Australia [94]. Like VEGF_121_, VEGF_111_ lacks extracellular matrix binding regions and, thus, is also freely diffusible [92], which is evident from the widespread vascular permeability induced by VEGF_111_ in comparison to VEGF_165_ [95]. Remarkably, this isoform is resistant to proteolytic cleavage and retains its complete biological activity upon exposure to plasmin, due to skipping of exon 5, which contains the residues Arg110-Ala111, the site of plasmin cleavage [96]. This is in contrast to all other isoforms, of which the biological activity is decreased upon exposure to plasmin [97]. VEGF_189_ and VEGF_206_ are the longest isoforms, containing both exons 6a and 7, with a strong affinity for heparin, being totally bound to ECM structures and less to the cell surface [77]. It is considered that, for this reason, those isoforms are less active than VEGF_121_ and VEGF_165_ [98]. Most VEGF-producing cells appear to preferably express VEGF_121_, VEGF_165_ and VEGF_189_, whereas VEGF_145_ and VEGF_206_ are comparatively rare, seemingly restricted to cells of placental origin [99, 100]. Recombinant VEGF_189_ and VEGF_206_ are unable to stimulate endothelial cell mitogenesis [77], since protein folding in these larger isoforms obscures regions responsible for receptor binding. The exon 6a-encoded sequence of VEGF_145_ confers affinity for heparin similar to that of the exon 7-encoded sequence of VEGF_165_. However, this sequence also mediates binding to components of the ECM that are independent of heparin or heparan sulphate. ECM-bound VEGF_145_ remains active as an endothelial cell mitogen [101]. Moreover, alternative splicing of the terminal exon, exon 8, gives rise to another isoform, VEGF-Axxxb, which has the same number of amino acids but different C terminal sequences. The differences between these two isoforms is based on deletion of 66 nucleotides from the beginning of exon 8 arising from a 3′ alternative splice site. [102] In addition to the VEGF-Axxxb isoform first identified, other isoforms have also been identified, like VEGF-A_121_b, which was confirmed to be present in normal human tissues and to bind VEGF receptors with an affinity similar to that of other VEGF isoforms, but to inhibit endothelial cell migration and to be protective to endothelial cells through VEGFR-2 activation [103].

The most important difference between the VEGF-Axxx and VEGFxxxb isoforms, however, is their effect on angiogenesis. While the VEGFxxx isoforms promote angiogenesis, VEGFxxxb is anti-angiogenic in nature, suggesting that an imbalance of the two could be crucial for the control over angiogenesis in healthy or pathological conditions [104].

## Detection of VEGFA, VEGFA_165_b and VEGFR in breast cancer tissues

**Fig. 4:**
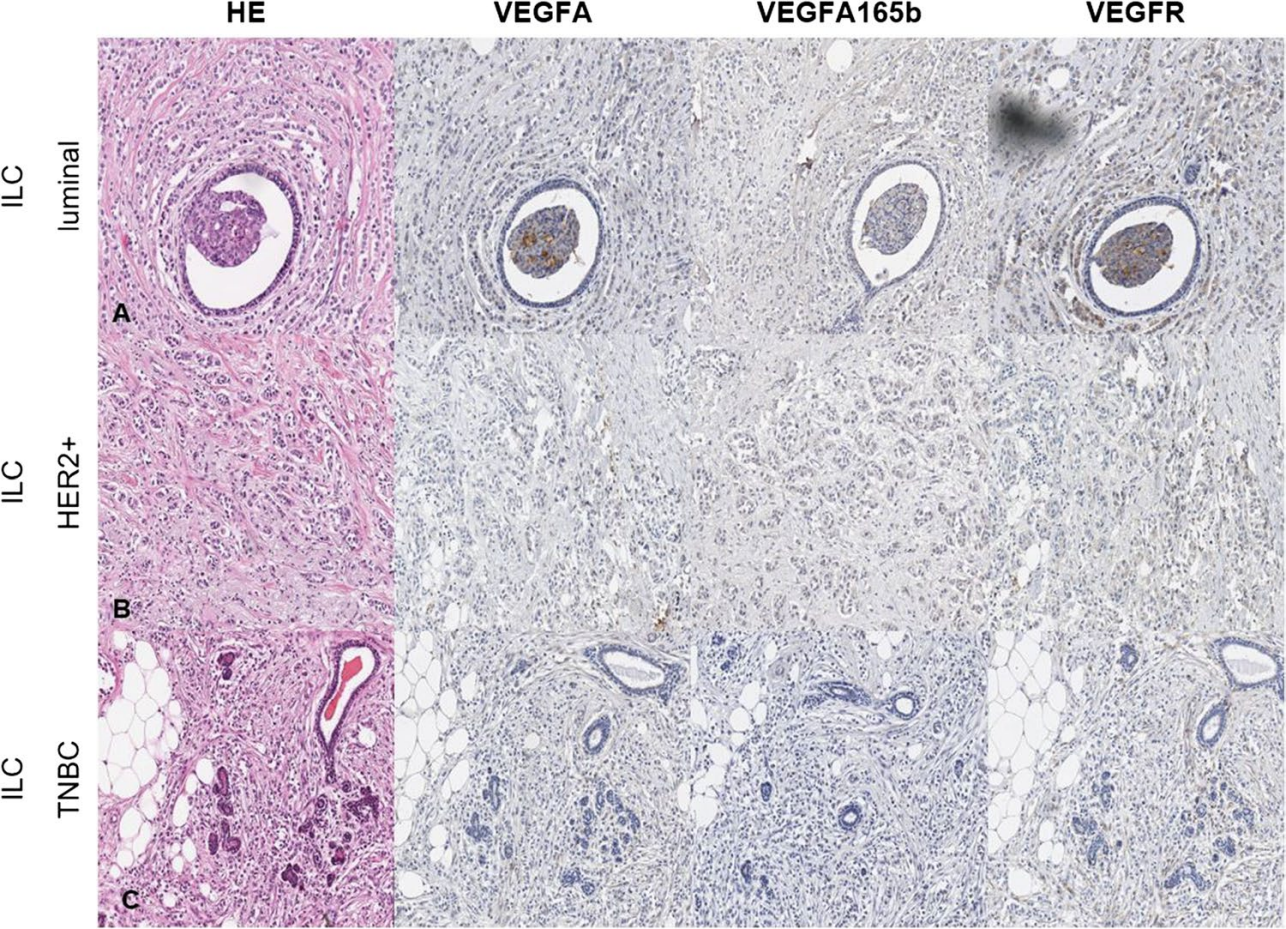
**Microscopic image of breast cancer ILC.** A: luminal BC, B: HER2 enriched BC. C: triple-negative BC

**Fig. 5:**
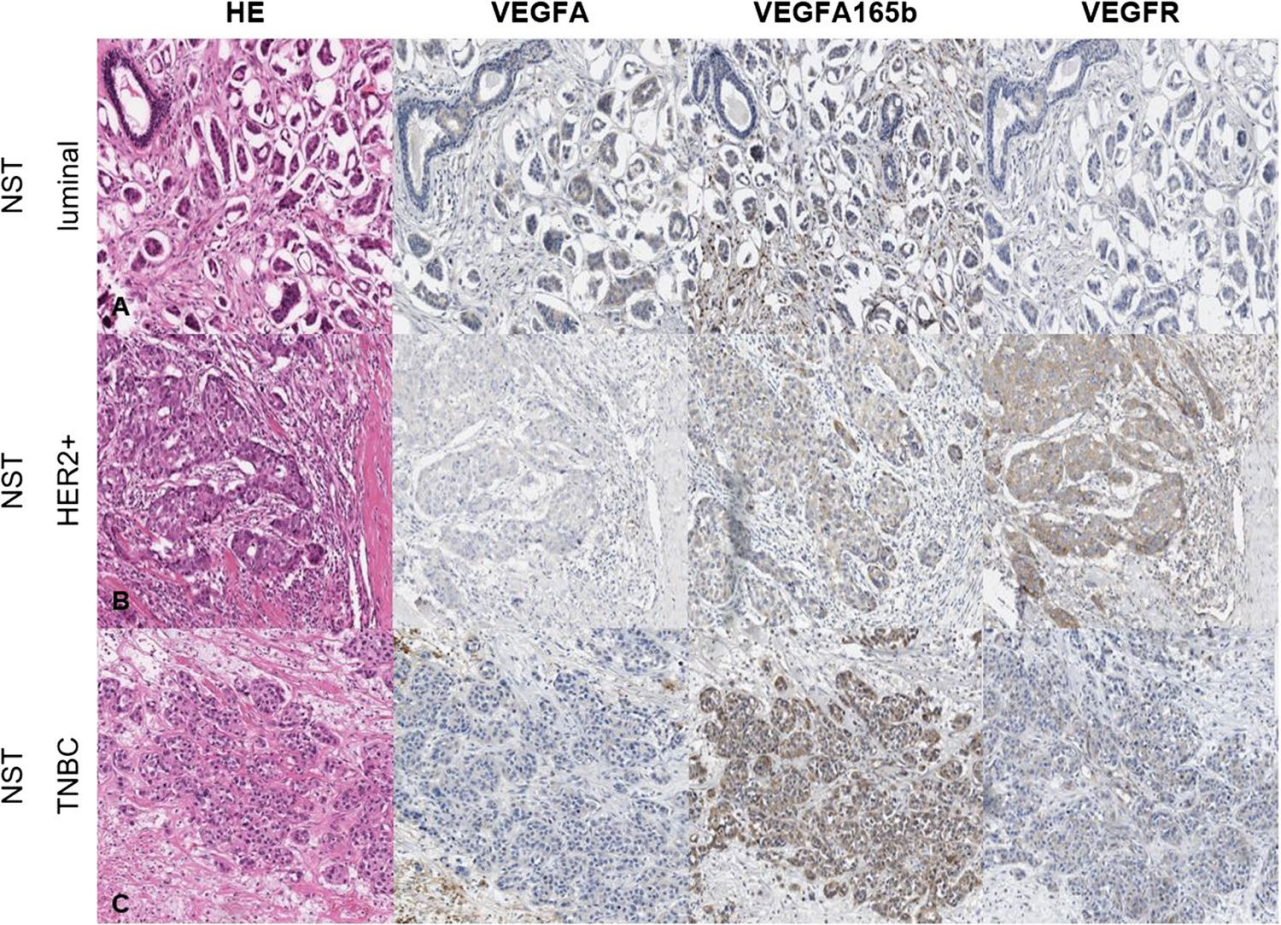
**Microscopic image of breast cancer NST.** A: luminal BC, B: HER2 enriched BC. C: triple-negative BC

Immunohistochemical detection of proteins is a common way to quantify biomarkers, which are useful for an optimal diagnosis and for a prediction of the efficacy of targeted therapies. To have a wide overview, we compiled HER2 enriched, luminal A and B and triple-negative formalin-fixed paraffin-embedded (FFPE) specimens of invasive breast cancer of no special type (NST) and of invasive lobular breast cancer (ILC). An anti-VEGFA antibody (VG-1 monoclonal, Abcam, 1:200), an anti-VEGF_165_b antibody (polyclonal, R&D System, 1:100) and an anti-VEGFR1 antibody (monoclonal Y103, Abcam, 1:100) were used for detection. Figure 4 shows microscopic images of hematoxylin and eosin, VEGFA, VEGF_165_b and VEGFR1 stained slides of breast cancer ILC tissues. Figure 5 shows similarly stained images of breast cancer NST tissues.

As expected, the cytoplasmatic VEGFA/_165_b expression is higher in invasive tumor cells than in normal tissues or stroma. So, we present examples showing that the detection of VEGF splice variants can be performed also at the protein level in formalin fixed tissues. However, no quantitative conclusions can be drawn from these images. Further studies to evaluate the expression of VEGF splice variants on a quantitative level are therefore needed.

## Balancing VEGF_165_ and VEGF_165_b

Due to the importance of VEGF_165_b, various studies have investigated its function and expression. It was found that VEGF_165_b is widely expressed in most healthy human tissues, such as retinal pigmented epithelia, smooth muscle, kidney, colon, lung, bladder, placenta and breast tissues [105], but also in human vitreous fluid [106], glomeruli [107, 108], podocytes [109] and aqueous humor [110], in some of them to a larger extent than VEGF_165_ [106, 111–114]. VEGF_165_b can also be found in blood, with levels consistent with known circulating VEGF levels [111, 115], as well as in the epidermis, but less in the dermis and blood vessels [116]. In the ovary it is expressed only in the theca externa, not in the theca interna [105]. It can be found in different states, i.e., bound as extracellular ligand or endothelial cell bound ligand [117], or as free VEGF in interstitial fluid.

A balance between VEGFxxx (pro-angiogenic) and VEGFxxxb (anti-angiogenic) proteins, being derived from the same gene, may play a crucial role in the control over angiogenesis in healthy tissues [111], whereas an imbalance could underpin pathological angiogenesis, leading to abnormalities such as dilated, tortuous or hyperpermeable vessels in the vascular network [111, 118] and impaired functions in the tumor vasculature [119]. The regulation of alternative splicing by growth and splicing factors plays an important role in determining the relative expression of pro- versus anti-angiogenic VEGF isoforms [111, 120, 121]. Examples of shifts from anti- to a pro-angiogenic VEGF isoforms have been seen in colorectal [122], kidney [102], breast [112] and prostate carcinomas [115], as well as in malignant melanoma [116] and pediatric neuroblastoma [118]. Diaz et al. [122] have not only been able to establish a connection between tumor growth and VEGF_165_b downregulation, but also between tumor stage, vascular invasion and lymph node metastasis. Although a study on breast cancer did not reveal a significant shift in the VEGF-A_165_b to VEGF-A ratio, it did find a statistically significant difference between breast cancer patients and a healthy control group. In addition to that, circulating VEGF-A_165_b was found to be significantly reduced in women with primary breast cancer at the time of diagnosis. Furthermore, the levels changed during adjuvant breast cancer treatment [123].

## How an imbalance of VEGF isoforms affects pathological conditions

Since VEGF isoform imbalances have been found in many tumors, this offers potential for treatment. Anti-angiogenic agents may improve the efficacy of drug delivery by normalizing this balance and, thus, tumor vasculature [119, 124]. Correspondingly, treatment is likely to be more effective in tumors with a high VEGF expression than in tumors with a low expression, as the latter have to rely on other pro-angiogenic factors for their growth [125]. According to Wu et al. [117, 126] most tissue-produced VEGF is consumed by local endothelial cells. As such, VEGF secretion in one tissue compartment has little or no effect on VEGF concentrations in other compartments. As mentioned above, VEGF_165_b is produced by podocytes [102], but interestingly it is only present in differentiated podocytes and not in undifferentiated ones, which suggests that the maturation of podocytes, endothelial cells and the glomerular basement membrane depends on the proportion of both isoforms [127]. VEGF_165_b significantly and dose dependently inhibits VEGF-165-mediated proliferation and migration of endothelial cells, and vasodilatation of mesenteric arteries in conditioned media [102, 128, 129]. Furthermore, it has been shown to inhibit hypoxia-driven angiogenesis in the retina and the growth of a variety of human tumor xenografts in mice [112, 114, 118, 130]. Recently, Zhang et al. found that VEGF_165_b and its mutant exhibits immunoregulatory functions, which suggests that it may serve as an immunomodulatory agent in cancer therapy, in addition to its anti-angiogenic abilities [131]. VEGF_165_b exerts its biological function by binding to its receptors, VEGFR-1 and VEGFR-2. It has been shown [117] that when VEGF_165_b increases, surface endothelial VEGFR-1 occupancy increases, whereas surface VEGFR-2 occupancy decreases and total VEGFR-2 occupancy remains constant, suggesting a shift in relative signaling by VEGFR-2 versus VEGFR-1. However, Mamer et al. recently found that VEGF-A_165_b selectively prefers VEGFR-2 binding with an affinity of 0.67 pM, while binding VEGFR-1 with a weaker affinity (KD = 1.4 nM) [132]. They showed that VEGF-A_165_b would preferentially bind VEGFR-2 (10 times stronger) than the VEGF-A_165_a variant, which binds VEGFR-1 with 3-orders of magnitude stronger than its anti-angiogenic counterpart. Woolard et al. [115, 133, 134] showed that VEGF_165_b inhibits VEGF165-mediated angiogenesis by blocking VEGF165-mediated VEGFR-2 phosphorylation. Kawamure et al. [134, 135] found that it served as a weak agonist of VEGFR-2 in vitro. One explanation for this discrepancy can be the alternative splicing of VEGF_165_b, in which the binding site to VEGFR-2, located in the proximal part of exon 8, is missing [136]. However, the results from Ganta et al. [24] indicate that the inhibition may be due to its ability to block VEGF165-mediated VEGF_165_R-1 activation rather than that of VEGFR-2.

## Possible outcomes of VEGF_165_b downregulation

Downregulation of VEGF_165_b has been observed in many tumors, as well as in diabetic retinopathy [106], Denys Drash Syndrome [137], retinal vein occlusion [138], glaucoma [139] and pre-eclampsia [140], while in other angiogenesis-related diseases, such as systemic sclerosis [141] and asthma [142], it is upregulated. Causative for VEGF_165_b downregulation may be overexpression of serine-arginine rich factor 1 (SRPK1), which leads to increased VEGF_165_a expression. As reported by Amin et al. [143], this overexpression may be due to a mutation in WT1, which leads to transcriptional repression as has been seen in patients with Denys Drash Syndrome [137], but also in the urogenital bud, where VEGF_165_b is expressed during embryonic development. Inhibition of VEGF_165_b results in abnormal ovariogenesis due to increased angiogenesis [144]. VEGF_165_b upregulation is due to stimulation of SRSF6 by activation of p38 mitogen-activated protein kinase (MAPK) and phosphorylation of Clk1/4 downstream of TGF-b [120], which can e.g. be observed in systemic sclerosis [141].

However, in certain diseased tissues no VEGF_165_b can be detected, as has been reported by Bates et al. [102], showing that the isoform was present in 17 of 18 normal kidney samples, but only in 4 of 18 matched malignant tissues. Another example for the absence of VEGF_165_b is malignant metastatic melanoma [116] with VEGF_165_b being present in most of the non-metastatic melanoma tissues and VEGF_165_ in just a few, similar to samples of the normal skin. According to this study, VEGF_165_b detection may identify patients at risk and even help in the prediction of metastasis. VEGF_165_b therefore plays an important role, not only in tumor development but also in its metastasis [76, 145, 146]. Nevertheless, there are still some tumors in which the balance of pro-angiogenic and anti-angiogenic VEGF isoforms seems to have a limited influence, such as on the development of parotid gland tumors [147]. In contrast, another recent study has shown that elevated levels of VEGF_165_b expression and a high VEGF_165_b/VEGF_165_ ratio correlates with the presence of lymph node metastases in non-small cell lung carcinoma [148]. Similar results were reported in another study showing that VEGFxxxb isoforms are upregulated in intraductal breast cancer [125]. Boudria et al. [148] were able to show that VEGF_165_b stimulates the proliferation and invasion of two lung tumor cell lines through a VEGFR/β1 integrin loop.

## Conclusion: VEGF_165_b as prognostic biomarker or therapeutic target?

It has been reported that the ratio VEGFxxx/VEGFxxxb has an effect on the sensitivity of tumors to bevacizumab, as both VEGF_165_ and VEGF_165_b can bind monoclonal antibody bevacizumab with a similar affinity [112]. Thus, the presence of VEGFxxxb can counteract the effect of this drug by reducing the amount of antibody available and, accordingly, less VEGFxxx can be inhibited. Hence, despite having a slower growth rate, tumors with high concentrations of VEGFxxxb may be more resistant to this therapy. Conversely, administration of additional VEGF_165_b appears to inhibit tumor growth [115, 149]. On the other hand, anti-VEGF_165_b antibodies in rodent developmental models seem to have pro-angiogenic effects and to contribute to the treatment of disorders in which VEGF_165_b is up-regulated [150]. Similar results have been reported by Manetti et al. [141], where a treatment combination of high-dose pro-angiogenic VEGF_165_ and anti-VEGF_165_b neutralizing antibodies improved systemic sclerosis. Similarly, Konopatskaya et al. [114] reported that injections of VEGF_165_b in an oxygen-induced retinopathy mouse model significantly reduced pre-retinal neovascularization, being associated with diabetic retinopathy. In addition, it has been found that inhibition of VEGFxxxb reduces glomerular endothelial and VEGF165-induced permeability in vitro [113]. Contrarily, Boudria et al. [148] found increased VEGF_165_b levels after treatment of lung adenocarcinoma cells with a high VEGF_165_b expression with bevacizumab, which indicates that there is crosstalk between VEGF_165_b, VEGFR-2 and β1 integrin proteins, promoting an invasive phenotype in these tumors. Next to monoclonal antibodies, promising results have recently been obtained with small molecule VEGF inhibitors like apatinib, a tyrosine kinase inhibitor, which can selectively inhibit phosphorylation of VEGFR-2. Chen et al. [151] showed that apatinib can enhance the anti-tumor effect of paclitaxel on triple negative breast cancer cells through the PI3K/p65/Bcl-xl signaling pathway, meaning that this combination may be a promising option for the treatment of this type of cancer. A novel oral tyrosine kinase inhibitor, surufatinib, which has a dual activity of anti-angiogenesis and immune regulation and simultaneously targets tumor angiogenesis (via VEGFR-1, VEGFR-2, VEGFR-3 and FGFR-1), has been approved in 2020 as a monotherapy for unresectable locally advanced or metastatic, progressive nonfunctioning, well differentiated (grade 1 or 2) extra-pancreatic neuroendocrine tumors (epNETs) in China, once again indicating that VEGF regulation may yield promising results in cancer treatment [152, 153].

In conclusion, further tissue-based research on VEGF_165_b and, more generally, on the various splice variants of VEGF may lead to significant advances in the design of targeted antibody-based (breast) cancer therapies.

## Data Availability

Not applicable.
